# The Hospital Library and Charities Bureau

**Published:** 1904-05-07

**Authors:** 


					HOSPITAL library and chari-
ties BUREAU.
tS 0f .ttle librarian are answered in this column free of
r^quiries to be answered promptly by post must
LiK a fee of 23. 6d. f ,,
announces the addition of 24 volumes of the
t"a to the Library for the use of members.
Gug Fire Drill.
!^6ts ?f "te Poor writes : I should be glad if any of 5 our
eeffiP!?Uld inform me how expert opinion can be obtained as to
?ncy of " fire drill" of officials in large institutions which
. ?4ji8!CpUlI1^er8 sick and infirm inmates.
v 'ion#,' ^ is proposed to publish an article thortlj on ro
Sllil .0ni Pire in Institutions. In the meanwhile the Editor will
0 receive information in reply to the above question.

				

